# Phase diagrams and dynamics of a computationally efficient map-based neuron model

**DOI:** 10.1371/journal.pone.0174621

**Published:** 2017-03-30

**Authors:** Mauricio Girardi-Schappo, Germano S. Bortolotto, Rafael V. Stenzinger, Jheniffer J. Gonsalves, Marcelo H. R. Tragtenberg

**Affiliations:** 1 Neuroimaging of Epilepsy Laboratory, McConnell Brain Imaging Center, McGill University, Montreal Neurological Institute and Hospital, H3A 2B4, Montreal, Quebec, Canada; 2 Departamento de Física, Universidade Federal de Santa Catarina, 88040-900, Florianópolis, Santa Catarina, Brazil; Consejo Nacional de Investigaciones Cientificas y Tecnicas, ARGENTINA

## Abstract

We introduce a new map-based neuron model derived from the dynamical perceptron family that has the best compromise between computational efficiency, analytical tractability, reduced parameter space and many dynamical behaviors. We calculate bifurcation and phase diagrams analytically and computationally that underpins a rich repertoire of autonomous and excitable dynamical behaviors. We report the existence of a new regime of cardiac spikes corresponding to nonchaotic aperiodic behavior. We compare the features of our model to standard neuron models currently available in the literature.

## 1 Introduction

Modeling the brain is not a simple task. Usually scientists opt for simple models that provide insights about the original phenomenon one is trying to study. On the other hand, these models may lack some fundamental features that would play an important role in the considered phenomenon, specially when studying complex systems like the brain. Nowadays supercomputers have allowed the Neuroscience community to propose *large-scale* models for either brain functions or brain electrophysiology [1–5].

Most of these models represent each neuron by a point in space that performs every computation expected to happen through the whole extended body of a real neuron. Herz *et al*. [6] have grouped single-neuron models in five broad categories (from detailed compartmental models to statistical simple models), but all of the considered models are generally described by ordinary differential equations (ODE). The more complex the model, the more details on how is the shape and dynamics (both in space and time) of the action potential propagating in the neuron membrane. Although very simple, single-compartment neurons are capable of performing some fundamental tasks of information processing in single-neurons [6].

Map-based neuron models have emerged in the last two decades as a new class of models that have simpler equations and are computationally efficient [7, 8]. Maps are discrete-time equations of continuous state variables, and map-based models describe the variation in time of the neuron membrane potential [9–16]. Thus all map-based neurons should fall in level three of Herz *et al*. [6] classification scheme (i.e. single-compartment models), given that there is no known map-based compartmental neuron model. Map-based neurons often have arbitrary discontinuities to model the abrupt voltage decrease after the action potential [13–15]. One of them is only an Euler-method solution for a simple non-linear integrate-and-fire ODE with unit time step (ts) [14]. Notice that maps are also used to model abstract properties of action potentials, such as the action potential duration [17] and the interbeat intervals [18] of cardiac cells, both following data-driven approaches.

Recently, a whole class of map-based neuronal models derived from the classical McCulloch-Pitts perceptron [19] has been described [8]. It consists of building up neuron models with recurrent dynamics from the simple perceptron until the desired dynamical complexity is reached. One of the richest neuron models of this class was introduced by Kinouchi & Tragtenberg [10] (KT). This model was originally proposed to study modulated magnetic systems, the axial-next-nearest-neighbor-Ising model [20].

The KT model is a two-dimensional map. The membrane potential is modeled by a hyperbolic tangent. With only three parameters and an external current it can present a large number of neuronal behaviors. However, in order to obtain neuronal bursting and cardiac spiking, the KT model was extended by Kuva *et al*. [11]. It was done by adding a third and slow variable *z*, thus creating the three-dimensional KTz model. Although both models are very interesting, the presence of the hyperbolic tangent demands substantial computational power when dealing with a large number of neurons.

We want to achieve the best trade-off between a good repertoire of membrane potential dynamical behaviors and computational efficiency. In this paper we approximate the original hyperbolic tangent gain function of the KT/KTz models by a logistic function while preserving all the other variables and parameters in both models. As a result we have two new sets of equations, the logistic KT (KTLog) and the logistic KTz models (KTzLog). Also, all the fixed points and the linear stability analysis become analytical. In Section 2, we describe the new governing equations and trace many bifurcation and phase diagrams both analytically and computationally. We then compare some of them with the original hyperbolic tangent version of the model. Finally, we compare our new model computational efficiency and dynamical behavior repertoire using frameworks proposed by Izhikevich [21] and Girardi-Schappo *et al*. [8] in Sections 3 and 4. We conclude the work in Section 5 discussing further applications of the current model.

The sigmoid functions used in both versions of the KT and KTz models are continuous functions, so the action potential reset is not forced into the system. As a consequence, KT/KTz spikes have their own rise and fall time scales which is useful for using more biologically inspired ways of coupling neurons in contrast to simple pulse coupling approaches [7, 8]. These intrinsic time scales make it possible for cardiac spikes to appear. Also, coupling currents enter in KT/KTz equations as additive terms inside the sigmoid function [22]. Large-scale models may be built up from such neurons using gap junctions or even chemical synapse maps [7, 8, 11] as simply as they can be built up from more complex conductance-based neuron models.

Using simple models of KTz family could facilitate the mathematical understanding of phenomena such as burst induced by subthreshold oscillations, cardiac arrhythmia or early afterdepolarization both studied *in vitro* and *in vivo* [23–25] or using conductance-based neurons [23, 25–28] which have huge parameter spaces. In fact, the study of many phenomena may be significantly enhanced by KTz family neurons due to the reduced dimension of its parameter space. In addition, KTz family and conductance-based models have such a similar way of coupling neurons that our map-based neurons would easily allow the study of compartmental neurons.

## 2 Models

The KT is a two-dimensional model defined by Eqs (1a) and (1b) below. The KTz is a tridimentional model defined by Eqs (1a)–(1c). In both models the gain function *f*(*u*) is a hyperbolic tangent. To obtain the KTLog and KTzLog models we approximate the hyperbolic tangent by its first order Taylor expansion, the logistic function *f*(*u*) = *u*/(1 + |*u*|):

$$x\left(t+1\right)=f\left(\frac{x\left(t\right)-Ky\left(t\right)+z\left(t\right)+H+I\left(t\right)}{T}\right),$$ (1a)

y(t+1)=x(t), (1b)

$$z\left(t+1\right)=\left(1-\delta \right)z\left(t\right)-\lambda \left(x\left(t\right)-x_{R}\right),$$ (1c)

In Eqs (1a)–(1c) *x*(*t*) is the actual membrane potential (in arbitrary units) of the cell at time *t* (measured in arbitrary time steps, ts), *y*(*t*) is a recovery variable, *z*(*t*) is the slow total ionic current (which may generate bursts and cardiac spikes) and *I*(*t*) is an arbitrary potential generated by external currents (due to synapses and/or electrodes). The *K* and *T* parameters control the fast spiking dynamics, the parameter *δ* is the inverse recovery time of *z*(*t*) and controls the refractory period, and *λ* and *x*_*R*_ adjust the slow spiking and bursting dynamics. Particularly, *λ* controls the damping of oscillations whereas *x*_*R*_ controls bursting duration. *H* is a bias membrane potential and *t* is the discrete time step. Every variable/parameter is in arbitrary scale.

The membrane potential of the cell represented by *x*(*t*) in Eq (1a) may display a wide variety of autonomous and excitable behaviors. Some examples are cardiac spikes, bursting, slow or fast spiking, types I and II excitability, rebound spikes and bursts, nerve blocking effect, accommodation, and many others. These and more behaviors will be discussed in Section 3. The following subsections are dedicated to explore the bifurcations of the model. We first present the phase diagrams to provide an overview of the dynamics. Following, we explicitly provide closed forms for the fixed points (FP) and their eigenvalues to trace specific bifurcation diagrams.

We separate the presentation of the model in two cases in order to simplify its understanding: (I) the two-dimensional KTLog model: *δ* = *λ* = *z*(0) = 0; and (II) the tridimensional KTzLog model: *H* = 0 and any *δ*, *λ* and *z*(0). Case I is the fast subsystem and case II is equivalent to taking case I and transforming its *H* parameter into the dynamical slow variable *z*(*t*), *H*^(case I)^ ≡ *z*(*t*), with arbitrary *δ*, *λ* and *x*_*R*_. Parameters *K* > 0 and *T* > 0 are assumed in this paper. The external current *I*(*t*) ≠ 0 is used to study the excitable behaviors of the model, but is kept zero for tracing all the bifurcation and phase diagrams.

### 2.1 Phase diagrams

A phase diagram contains the stability limits of the model attractors in the parameter space. It gives a general picture of the frontiers between stable attractors of the model, be they FP or oscillatory attractors (OA). It is worth noticing that both cases of our map present three different types of OA: periodic, quasi-periodic and chaotic. Chaotic attractors are going to be discussed in Section 3.

A FP (x*, y*, z*) is the point that does not change with map iteration, such that

$$\begin{array}{c} \left[x(t+1),y(t+1),z(t+1)\right]=\left[x(t),y(t),z(t)\right]=\left(x^{*},y^{*},z^{*}\right). \end{array}$$ (2)

A FP may also be referred to as a 1-cycle, because the map iteration exactly repeats after every 1 ts. Any periodic *Q*-cycle (*Q* > 1) may be determined similarly by [*x*(*t* + *Q*), *y*(*t* + *Q*), *z*(*t* + *Q*)] = [*x*(*t*), *y*(*t*), *z*(*t*)]. However, the *Q*-cycle regions in the phase diagram of case I are computationally calculated due to the variety of possible OA in the model.

For a given FP (x*, y*, z*), the stability limit is obtained using the condition

$$\begin{array}{c} \max\left\{|\Lambda_{1}\left|,\right|\Lambda_{2}\left|,\right|\Lambda_{3}|\right\}=1, \end{array}$$ (3)

where Λ_*i*_ are the eigenvalues of the FP. Eq (3) yields an equation for x* over the stability limit. Therefore, the stability limit curve may be obtained by applying the x* thus calculated to Eq (2) and isolating the parameter of interest as function of the other parameters. Notice that for case I there are only two eigenvalues in Eq (3). Also, both y* and z* are linear functions of x* so it is sufficient to calculate x* to fully determine the FP.

A given OA corresponds to a neuronal oscillatory behavior such as fast spiking (FS), bursting (BS) or cardiac spiking (CS). We use the interspike interval (ISI) distribution, P(ISI), to further distinguish between oscillatory behaviors for case II. We give details about the ISI distribution in the supporting S1 File. We also calculate the maximum amplitude, *A*, of the oscillations,

$$\begin{array}{c} A=\left|\max_{t}\left\{x(t)\right\}-\min_{t}\left\{x(t)\right\}\right|\;, \end{array}$$ (4)

where *x*(*t*) is a given OA, in order to identify pure subthreshold oscillations (SO) phases.

#### 2.1.1 Case I

The neuron has three parameters (*K*, *T* and *H*). We then trace diagrams *K* × *T* for *H* = 0 and then *T* × *H* for many *K*. We use the stability limit condition given in Eq (3) in order to derive the phase boundaries. The FP and its eigenvalues are given in the next subsection [Eq (15)] together with the description of each bifurcation.

**Fig 1:**
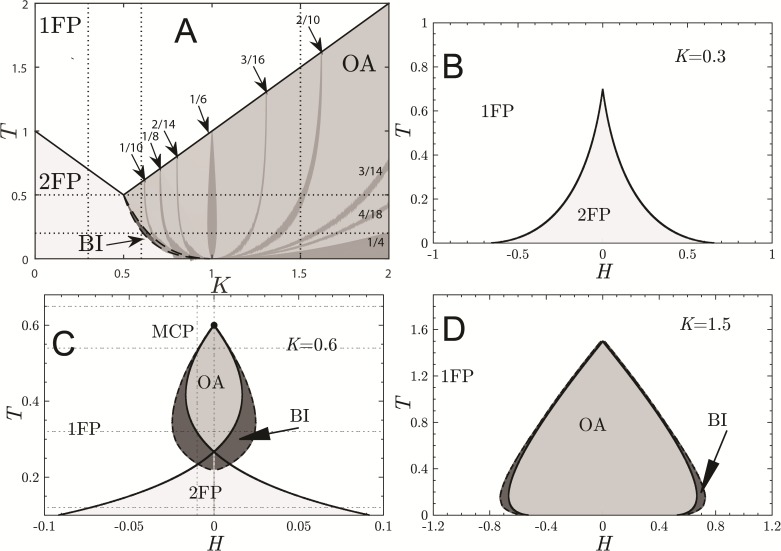
Phase diagrams of the KTLog model (case I). Panel A: *T* × *K* plane for *H* = 0 (vertical dashed lines are the selected *K* values for panels B, C and D); Solid lines are supercritical pitchfork bifurcation [1FP↔2FP, Eq (5)] or supercritical Neimark-Sacker bifurcation [1FP↔OA, Eq (6)], rightmost dashed line of the BI region is a subcritical Neimark-Sacker bifurcation [2FP→OA, Eq (7)] and leftmost dashed line of the BI region is the stability limit of the OA region. Panel B: *T* × *H* plane for *K* = 0.3 (valid for *K* ≤ 0.5); Panel C: *T* × *H* plane for *K* = 0.6 (valid for 0.5 < *K* < 1); Panel D: *T* × *H* plane for *K* = 1.5 (valid for *K* ≥ 1). Panels B–D: 2FP↔1FP are saddle-node bifurcations and dashed lines are subcritical Neimark-Sacker bifurcations; solid lines are given by Eq (8). The top point (multicritical points, MCP) in panels C and D are supercritical Neimark-Sacker bifurcations. The OA region contains many periodic Arnold tongues labeled by their winding numbers.

There are three analytically determined stability limits for *H* = 0 (depicted in Fig 1A):

T=1−K,for K≤0.5, (5)

T=K,for K>0.5, (6)

$$T=\frac{1}{K}+K-2,\text{for }0.5\leq K\leq 1.$$ (7)

where Eq (5) separates a region of one stable fixed point (1FP) from two stable fixed points (2FP) via a supercritical pitchfork bifurcation, Eq (6) separates 1FP from an oscillatory attractor region (OA) by a supercritical Neimark-Sacker bifurcation and Eq (7) separates 2FP from OA region through a subcritical Neimark-Sacker bifurcation. The curve given by Eq (7) is the rightmost dashed line of the bistability (BI) region in Fig 1A. These bifurcations are going to be further discussed in the next section. The OA→2FP stability limit has been determined by map iteration and is the leftmost dashed curve of the BI region in Fig 1A. The BI region contains at least two kinds of stable attractors: OAs and FP.

The OA region is filled up with Arnold tongues, each corresponding to a different periodic OA. The labels for these tongues in Fig 1A are winding numbers *w* ≡ *P*/*Q*, where *Q* is the attractor period (i.e. the amount of time steps the map takes to exactly repeat itself) and *P* is the amount of complete oscillations inside a period *Q*. The OAs in this region are born in a supercritical Neimark-Sacker bifurcation (the line *T* = *K*_*c*_) over which |Λ_+_| = |Λ_−_| = 1. Thus, we may determine the labels simply by $w=\frac{1}{2\pi }\arccos\left(\frac{1}{2K_{c}}\right)$.

The tongues are thinner than in the original hyperbolic tangent model [10] and they correspond to the periodic attractors in the bifurcation diagrams of the next subsection. The overall structure of the diagram is preserved by the logistic function approximation. Details about the algorithm used to determine the tongues are given by Tragtenberg & Yokoi [20].

Parameter *H* is a third axis going out of the paper in Fig 1A. Then for *H* ≠ 0, we have three qualitatively distinct phase diagrams (see Fig 1B–1D) that correspond to the stability limits of Eqs (5), (6) and (7): *K* < 0.5; 0.5 ≤ *K* < 1 and *K* > 1. Vertical dashed lines in Fig 1A indicate *K* values used to trace the other three phase diagrams in the figure. The solid lines in Fig 1B–1D are the FP stability limits:

$$\begin{array}{c} H=\left\{\begin{array}{cc} \left(\sqrt{(1-K)T}+K-1\right)x^{*}\;, & \text{for }K<0.5,\\ \left(\sqrt{KT}+K-1\right)x^{*}\;, & \text{for }K\geq 0.5, \end{array}\right. \end{array}$$ (8)

derived from Eq (2), where x* are the FP values calculated from Eq (3):

$$\begin{array}{c} x^{*}=\left\{\begin{array}{cc} \pm \left(1-\sqrt{\frac{T}{1-K}}\right)\;, & \text{for }K<0.5,\\ \pm \left(1-\sqrt{\frac{T}{K}}\right)\;, & \text{for }K\geq 0.5, \end{array}\right. \end{array}$$ (9)

We have two distinct solutions for Eqs (8) and (9) because the eigenvalues change from real to complex on *K* = 0.5. Eq (9) holds only over the bifurcation line, Eq (8). The full expression for the FP will be derived in the next subsection.

The curves defined by Eq (8) are vertical slices of the three-dimensional parameter space (perpendicular to *K*-axis and parallel to *T*-axis in Fig 1A). The dashed lines in Fig 1C and 1D are the stability limits of the OA phase and have been determined by map iteration. The OA phase is also full of Arnold tongues which are not shown for simplicity. The phase diagrams in Fig 1 give us a general picture of all the bifurcations of the model (some of which will be further detailed in the next subsection).

There is again a striking similarity between our approximation and the original model by Kinouchi & Tragtenberg [10] with two important differences: first, the two multi-critical points (MCP) from the original model coalesced into only one MCP (Fig 1C), sharpening the top-most part of the curves into a cusp-like form; second, the original supercritical Neimark-Sacker bifurcation has now turned into subcritical for *K* > 1 (Fig 1D) and only the beak of this curve corresponds to a supercritical Neimark-Sacker bifurcation.

Comparing the diagrams in Fig 1, we conclude that the richest region in terms of dynamical behavior is within 0.5 < *K* < 1 because it presents all the phases of the model. Thus, we will study the 3-dimensional model only inside this range, even though other as interesting behaviors might be found for other values of *K*.

#### 2.1.2 Case II

The variable *z* brings three new parameters to analyze (*δ*, *λ* and *x*_*R*_). We trace the *x*_*R*_ × *T* diagram for fixed *δ* = *λ* = 0.001 and *K* = 0.6 applying the interspike interval (ISI) method described in Methods section to determine the phases. It is possible to trace diagrams using the ISI method to any combination of parameters, but we will present only this case in order to compare with the diagram of the hyperbolic tangent model presented by Kuva *et al*. [11].

**Fig 2:**
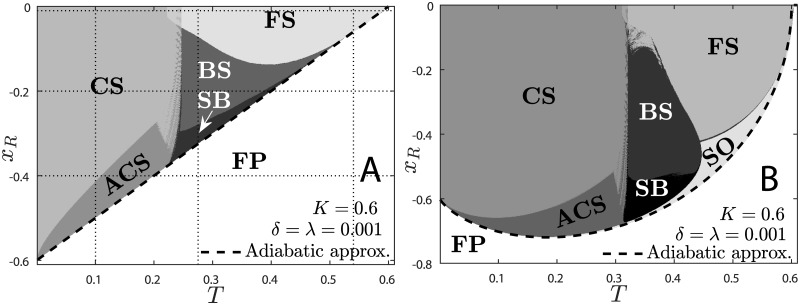
Phase diagram *x*_*R*_ × *T* for the logistic and the tanh KTz models. Panel A: ISI distribution method applied to the KTzLog model (case II). Panel B: ISI method applied to hyperbolic tangent KTz model (compare to the one presented in [11]). Phases are displayed with different shades of gray. There is a bistable region on the boundary between FS and CS. The characteristic behavior of the map inside the *dust* between CS and BS is an infinite switching between cardiac spikes and bursting. Dashed line is the result of the quasi-static approximation. Subthreshold oscillations (SO) are also present in panel A, but within a very thin region under the dashed line. Slow bursting and bursting are separated arbitrarily to highlight that the map presents bursts with many interburst interval. Vertical and horizontal dotted lines correspond to values chosen to plot Fig 5.

Fig 2 presents the phases determined by the ISI scheme described in Methods. The typical behavior of each phase is depicted in Fig A in S1 File. We plot the diagram for case II on panel A and compare it with the same method applied to the tanh KTz. Panel B can also be compared to the diagram created using only map iteration in [11].

The FP stability limit may be easily calculated via an adiabatic approximation [considering that *z*(*t*) varies very slowly compared to *x*(*t*)]. It consists in taking *H* = z* = (*x*_*R*_−x*)*λ*/*δ* in case I and solving for the stability limits [Eqs (8) and (9)] in the plane *x*_*R*_ × *T* [29]. We obtain (for 0 < *δ* = *λ* ≪ 1):

$$\begin{array}{c} x_{R}=\pm \left(K-T\right). \end{array}$$ (10)

Eq (10) is plotted as a dashed line in Fig 2A. This curve is way simpler than the one obtained with the hyperbolic tangent model (dashed line in Fig 2B) [29]. In principle, the stability limits of the KTzLog model could be precisely calculated for any *δ* and *λ* range, due to its analytical eigenvalues (see next subsection). The curve given by Eq (10) approximates [O(δ)] the line of a supercritical Neimark-Sacker bifurcation between the FP and subthreshold oscillations (small amplitude OAs) in the plane *x*_*R*_ × *T*.

Both diagrams present the same autonomous OA phases: cardiac spiking (CS), fast spiking (FS), bursting (BS) and subthreshold oscillations (SO). These behaviors are depicted in Fig 7I, 7G, 7J and 7M, respectivelly. The SO region is very narrow for the logistic case and is better identified by the amplitude of oscillations presented together with the bifurcation diagrams. Slow bursting (SB) has been separated from regular bursting using an arbitrary threshold only to emphasize that the interburst interval (IBI) diverges quickly near the bifurcation, as will be discussed ahead.

The ISI method provides an automated way to separate phases and also sheds light on structures and bifurcations which are very difficult to find via map iteration. Two features were identified using this method: the aperiodic cardiac spiking (ACS) region and the *dust* in the phase diagram between CS and BS. Both are also present in the original tanh version, but had not been previously identified using map iteration [11].

The ACS region is composed of aperiodic non-chaotic cardiac spiking attractors (see Fig A, panel D, in S1 File). The periods of these OAs are distributed within a broad skewed bell-shaped distribution. The OA over the *dust* region between CS and BS is characterized by constant switching between CS and BS behavior (see Fig 7L and right-hand plot of Fig 6C). The dust region comprises very chaotic behaviors and might have a fractal *shrimp* structure in the plane *x*_*R*_ × *T*, similarly to the parameter space of other oscillators [30, 31]. This will be investigated in forthcoming works.

### 2.2 Fixed points and bifurcations

#### 2.2.1 Case I

Making use of the logistic function along with the FP definition, it is possible to write Eqs (1a) and (1b) as:

$$\begin{array}{c} \begin{array}{cc} x^{*}= & \frac{x^{*}-Ky^{*}+H}{p},\\ y^{*}= & x^{*}, \end{array} \end{array}$$ (11)

where *p* ≡ *T* + |*p*_0_| and *p*_0_ ≡ (1 − *K*)x* + *H*.

Reorganizing the terms in Eq (11), we reach:

$$\begin{array}{c} {\left(x^{*}\right)}^{2}+\frac{K+T+sH-1}{s(1-K)}x^{*}-\frac{H}{s(1-K)}=0, \end{array}$$ (12)

with solutions:

$$\begin{array}{c} x_{\pm }^{*}=\frac{s}{2(1-K)}\left[1-K-T-sH\pm \sqrt{{\left(1-K-T+sH\right)}^{2}+4sHT}\right], \end{array}$$ (13)

where *s* ≡ |*p*_0_|/*p*_0_ = ±1. Eq (13) provides four FP from which we keep only the ones that satisfy: (a) $x^{*}\in R$, (b) |x*| ≤ 1 and (c) the implicit inequality in |*p*_0_| on *s* definition, i.e. *p*_0_ > 0 for *s* = +1 and *p*_0_ < 0 for *s* = −1.

The eigenvalue, Λ, equation is:

$$\begin{array}{c} \Lambda^{2}-\frac{T}{p^{2}}\Lambda +\frac{KT}{p^{2}}=0, \end{array}$$ (14)

with solutions:

$$\begin{array}{c} \Lambda_{\pm }=\frac{T}{2p^{2}}\left(1\pm \sqrt{1-\frac{4Kp^{2}}{T}}\right). \end{array}$$ (15)

The dependence of Λ on $x_{\pm }^{*}$ and *H* is implicitly inside *p*. These eigenvalues assume a complex value for *K* > 0.5, when *H* = 0, allowing for the appearance of OAs.

**Fig 3:**
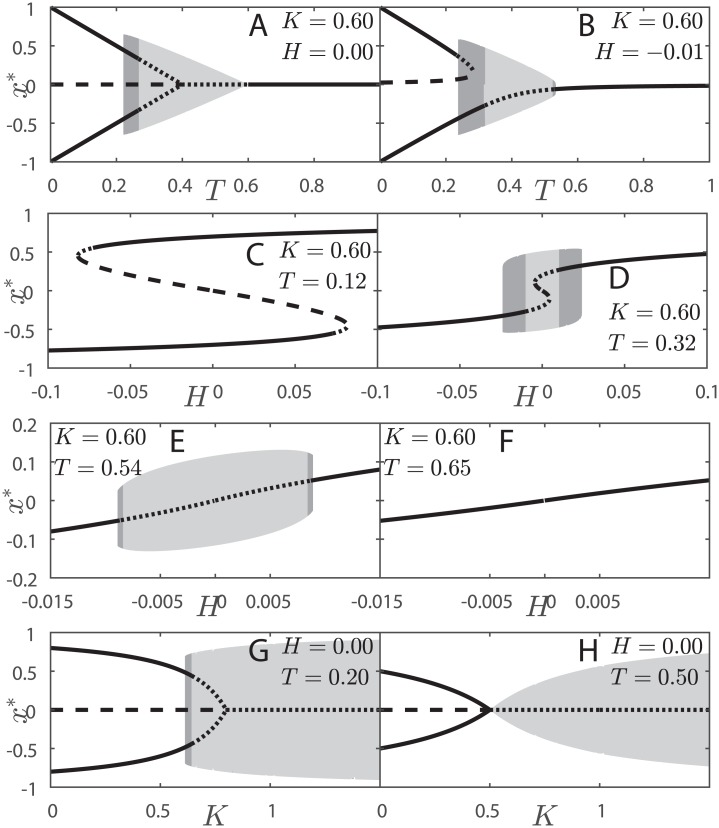
Bifurcation diagrams of the KTLog model. Panels A, B: bifurcation over *T* for fixed *K* = 0.6. (A) *H* = 0.0 and (B) *H* = −0.01. Panels C–F: bifurcation over *H* for fixed *K* = 0.6. (C) *T* = 0.12, (D) *T* = 0.32, (E) *T* = 0.54, (F) *T* = 0.65. Panels G, H: bifurcation over *K* for fixed *H* = 0.0. (G) *T* = 0.2, (H) *T* = 0.5. Eqs (13) and (15) give stable FPs (—), unstable FPs (⋯) and saddle FPs (---); and Eq (4) gives the amplitude of OAs (height of the filled up area). Dark grey: coexistence of stable OAs and at least one stable FP; Light gray: coexistence of stable OAs and unstable FPs.

We plot the bifurcation diagrams (Fig 3A–3H) versus each of the three parameters of the map, *K*, *T* or *H*. As usual, in these diagrams the stable FPs (|Λ_±_| < 1) are plotted with solid lines whereas the unstable FPs (|Λ_+_| > 1 and |Λ_−_| > 1) are plotted with dotted lines. Saddle FPs (at least one of the eigenvalues has a different sign than the others) are plotted with dashed lines.

The gray regions in Fig 3A–3H correspond to OAs obtained by map iteration. The heights of the colored areas give the maximum amplitude of the OAs, calculated with Eq (4). These regions are filled up with many periodic attractors, or *Q*-cycles, windows. Most of these windows are too narrow to be perceived, so we do not show them. In Section 2.1, we trace the phase diagram of the model’s case (I) with *H* = 0, clearly showing its periodic attractors.

The dark gray region in these bifurcation diagrams represent co-stability of OAs with at least one stable FP. These regions’ internal borders then present subcritical Neimark-Sacker bifurcations (also known as Andronov-Hopf bifurcations for continuous time systems).

The bifurcation of x* over *T* parameter in Fig 3A and 3B changes from a pitchfork-like bifurcation in A for *H* = 0 to a cusp-like catastrophe in B when |*H*| > 0. In the case of *H* < 0 (*H* > 0), the bottom (top) branch gets disconnected from the top (bottom) branch in panel B. The behavior depicted in this figure is valid for 0.5 < *K* < 1. For 0 < *K* < 0.5, the OAs region vanishes, whereas for *K* > 1 the 2FP region vanishes. The behavior of the map is symmetrical in *H*. This fact is evident in the phase diagrams of Section 2.1.

Fig 3C–3F shows the cusp catastrophe surface projection on the x* × *H* plan (two saddle-node bifurcations between unstable and saddle equilibria). The hysteresis curve in C becomes a single stable FP in F with increasing *T*, passing through a region of stable OA. In these panels, the FP loses stability via a subcritical Neimark-Sacker bifurcation, but the OAs that emerge in Fig 3C are only unstable and bifurcate by touching the saddle equilibrium as |*H*| increases. This figure is valid for 0.5 < *K* < 1. The OA regions vanish for *K* < 0.5 and, on the other hand, the 2FP region vanishes for *K* > 1. The value of the the parameters *K*, *T* and *H* at which the two FPs coalesce into one is easily obtained analytically using the stability diagrams which we shall present in Section 2.1.

Fig 3G and 3H shows the bifurcation of x* according to *K* parameter for *H* = 0 and two different *T*. As *T* is increased, the OAs region decouples from the two stable FPs (2FP) region, changing from a subcritical [Fig 3G] to a supercritical [Fig 3H] Neimark-Sacker bifurcation for *T* ≥ 0.5. For *H* ≠ 0, the symmetry is broken similarly to the bifurcation over *T* in Fig 3B, i.e. either the top (*H* > 0) or bottom (*H* < 0) FP survives and the other vanishes in a Saddle-Node bifurcation. Also, OAs exist only within a certain range of *H* close to zero and any |*H*| > 0 turns the supercritical Neimark-Sack bifurcation into subcritical. Again, there is symmetry in *H* → −*H*.

#### 2.2.2 Case II

The FP equation for case II is easily derived from Eq (12), one should just replace *H* by z* = (*x*_*R*_−x*)*λ*/*δ*, resulting in the equation

$$\begin{array}{c} s\left(1-K-\alpha \right){\left(x^{*}\right)}^{2}+\left(T+sx_{R}\alpha -1+K+\alpha \right)x^{*}-\alpha x_{R}=0, \end{array}$$ (16)

where we define *α* ≡ *λ*/*δ*. The solution is simply:

$$\begin{array}{c} x_{\pm }^{*}=\frac{s}{2(1-K-\alpha )}[1-K-T-\alpha \left(sx_{R}+1\right)\pm \sqrt{{\left(T+K+\alpha -1\right)}^{2}+2sx_{R}\alpha \left(1-K-\alpha +T\right)+\alpha^{2}x_{R}^{2}}]. \end{array}$$ (17)

The FPs given in Eq (17) exist only if they satisfy the same conditions listed for Case I, replacing *H* by z* in the third condition, namely $x^{*}\in R$, |x*|≤1 and (1 − *K*)x* + z* > 0 for *s* = +1 and (1 − *K*)x* + z* < 0 for *s* = −1.

The Jacobian eigenvalues polynomial is given by:

$$\begin{array}{c} p^{2}\Lambda^{3}-\left[T+\left(1-\delta \right)p^{2}\right]\Lambda^{2}++T\left(K+\lambda +1-\delta \right)\Lambda -KT\left(1-\delta \right)=0, \end{array}$$ (18)

recalling that *p* = *T* + |x* − Ky* + z*|. It has solutions

$$\begin{array}{c} \begin{array}{cc} \Lambda_{1} & =B+C-\sqrt[{3}]{2}D,\\ \Lambda_{2} & =B-\frac{1-\sqrt{3}i}{2}C+\frac{1+\sqrt{3}i}{\sqrt[{3}]{4}}D,\\ \Lambda_{3} & =B-\frac{1+\sqrt{3}i}{2}C+\frac{1-\sqrt{3}i}{\sqrt[{3}]{4}}D, \end{array} \end{array}$$ (19)

where the coefficients *B*, *C* and *D* are given:

$$\begin{array}{c} \begin{array}{cc} B= & \frac{T-\left(\delta -1\right)p^{2}}{3p^{2}},\\ C= & \frac{\sqrt[{3}]{R+\sqrt{4U^{3}+R^{2}}}}{3\sqrt[{3}]{2}p^{2}},\\ D= & \frac{U}{3p^{2}\sqrt[{3}]{R+\sqrt{4U^{3}+R^{2}}}},\\ U= & 3p^{2}T\left(K+\lambda -\delta +1\right)-{\left[\left(\delta -1\right)p^{2}-T\right]}^{2},\\ R= & 2T^{3}-3\left(\delta -1\right)p^{4}T\left(\delta -1+6K-3\lambda \right)+3p^{2}T^{2}\left(\delta -1-3K-3\lambda \right)-2{\left(\delta -1\right)}^{3}p^{6}. \end{array} \end{array}$$ (20)

**Fig 4:**
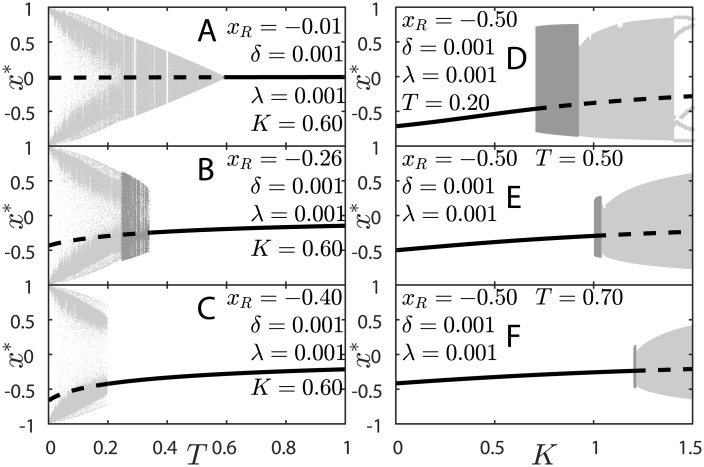
Bifurcation diagrams of the KTzLog model. Panels A–C: bifurcation over *T* for fixed *K* = 0.6 and *δ* = *λ* = 0.001. (A) *x*_*R*_ = −0.01, (B) *x*_*R*_ = −0.26, (C) *x*_*R*_ = −0.4. Panels D–F: bifurcation over *K* for fixed *x*_*R*_ = −0.5 and *δ* = *λ* = 0.001. (D) *T* = 0.2, (E) *T* = 0.5, (F) *T* = 0.7. Eq (17) gives stable FPs (—) and unstable FPs (---) and Eq (4) gives the amplitude of OAs (height of the filled up area). In panel D, instead of the height of the attractor for *K* ≲ 1.5, the actual attractor is plotted as an example of a periodic OA. Dark grey: bursting; Light gray: fast spiking or cardiac spiking.

We plot the bifurcation diagrams of the model in Fig 4A–4F, with FPs given by Eq (17) and FPs stability given by Eq (19). Solid lines are stable FPs and dashed lines are saddle FPs. OAs again reside in the colored areas.

For Fig 4A–4C, the colored area contains the points *x*(*t*) of the actual OA attractor for each *T* iterated for 10^6^ ts. If we had iterated the map for more time steps, the attractors would have more densely distributed points for each *T*. For Fig 4D–4F, the colored areas’ heights give the amplitude of the OAs, Eq (4). BS lies in the dark gray region and FS or CS lie in the light gray region. The horizontal gaps are periodic attractor windows (corresponding to Arnold tongues in the phase diagrams of the previous subsection).

The *z* slow oscillation makes the 2FP region from the 2-dimensional model disappear, making it a single FP region. The values of *x*_*R*_, *λ* and *δ* determine the steady value of z* = (*x*_*R*_−x*)*λ*/*δ*, which is equivalent to *H* in case I. Thus, the described behavior is symmetrical in *x*_*R*_, although the FPs would be positive.

In Fig 4A–4C, the FP loses stability via supercritical Neimark-Sacker bifurcation to, initially, SO. The 2FP region that is present in case I generates the CS region for case II. If one considers *z* varying very slowly (a quasi-static approximation), then a CS may be regarded as a slow switching between two stable FP. This statement can be verified by comparing Figs 3A, 3B and 4A–4C and noticing the high density of attractor points in CS region for case II where there were previously the two FPs of case I. The high density of the attractor close to the case I FP highlights the presence of a Saddle-Node bifurcation nearby (see Fig 3C) and is known as attractor ruins or ghosts [32, 33].

In Fig 4D–4F, the FP also loses stability via a supercritical Neimark-Sacker bifurcation to a SO regime. The SO grows in amplitude according to a power law as *K* is increased and give rise to the BS region. The BS region shrinks as *T* increases. The OA amplitudes decrease as *T* increases. The decreasing of amplitude of OAs may also be explained by looking to case I: For *H* = 0 and *T* = *K* (*K* > 0.5) there is a supercritical Neimark-Sacker bifurcation in case I (see Figs 1A and 3A), therefore for |*x_R_*λ/δ| ≳ 0 and *T* ≲ *K* the amplitude of OAs should be small. In panel D, particularly, there is a large periodic window in the right-hand side of the OA region, displaying a 5-cycle attractor.

The FS and BS regions get extinguished as |*x*_*R*_| increases (see phase diagram in Fig 2A). The bifurcations’ description presented so far for case II is valid for 0.5 < *K* < 1. For 0 < *K* < 0.5, there is only FP and CS behavior whereas for *K* > 1 there is no CS, but FS and FP. The same pattern repeats for *x*_*R*_ > 0, but the oscillations occur around the symmetrical positive FP. The general picture of the bifurcations for all *K* values is better observed in Fig 1.

**Fig 5:**
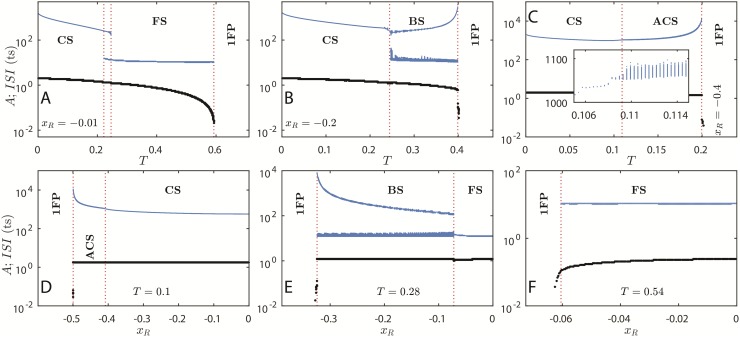
ISI bifurcation diagrams and OA amplitude. ISI is displayed in the top curves and OA amplitude in the bottom ones (circles) for *K* = 0.6 and *δ* = *λ* = 0.001. Panels A–C: *ISI* × *T* for *x*_*R*_ = −0.01 (A), *x*_*R*_ = −0.2 (B) and *x*_*R*_ = −0.4 (C); inset: detail of the plot in the frontier between cardiac spikes and aperiodic cardiac spikes; notice how the ISI goes from a characteristic value to several distinct values. Panels D–F: *ISI* × *x*_*R*_ for *T* = 0.1 (D), *T* = 0.275 (E) and *T* = 0.54 (F). Vertical dotted lines highlight bifurcations. The amplitude of limit cycles, Eq (4), has discontinuities at every bifurcation. These discontinuities tend to disappear as |*x*_*R*_|→0 because then the model approaches a supercritical Neimark-Sacker bifurcation. The amplitude also highlights the presence of subthreshold oscillations in the transition from FP to BS or to ACS.

**Fig 6:**
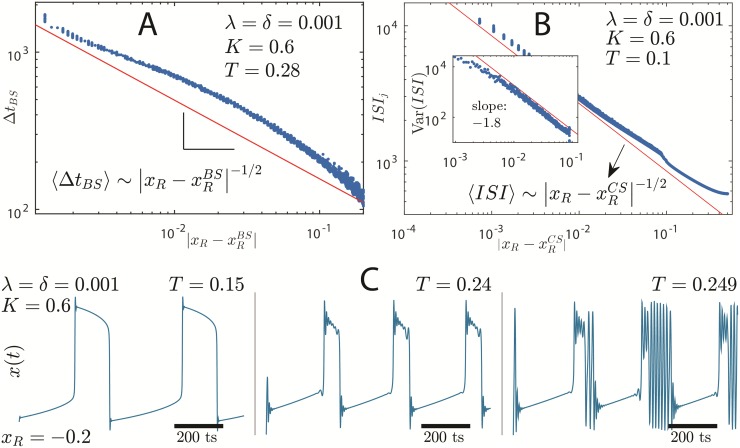
Infinite period bifurcation, blue-sky catastrophe and CS→BS transition. Panel A: Burst duration, Δ*t*_*BS*_, as a function of $|x_{R}-x_{R}^{BS}|$, where $x_{R}^{BS}$ is the bifurcation point in which fast spiking begins through a blue-sky catastrophe [34]. The inverse square root is only valid very close to the bifurcation. Panel B: Period of oscillations, *ISI*_*j*_, as function of $|x_{R}-x_{R}^{CS}|$, where $x_{R}^{CS}$ is the bifurcation point given by Eq (10) in the quasi-static approximation. Notice how 〈*ISI*〉 goes to infinity as the system gets closer to the bifurcation according to the inverse square root. This figure presents the same data as in Fig 5D. A small beak near $|x_{R}-x_{R}^{CS}|\approx 10^{-1}$ separates aperiodic (left) from periodic (right) cardiac spiking. Panel B inset: variance of *ISI*, a power law with slope −1.8. Panel C: Transition from CS to BS for increasing *T* and fixed *K* = 0.6, *δ* = *λ* = 0.001 and *x*_*R*_ = −0.2. Notice as slow oscillations lose stability going through a behavior of mixed cardiac spikes and bursting. The shape of action potential in panel C, middle, resembles that of a early depolarized action potential [28].

**Fig 7:**
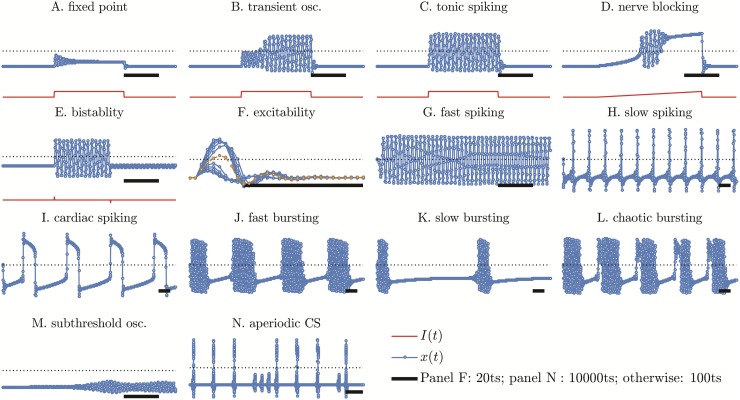
Excitable KT and autonomous KTz behaviors. Panels A to F: case I specific excitable behaviors. Remaining panels: case II autonomous oscillations. Parameters are given in Table A in S1 File.

We calculate ISIs in order to reveal the inner structure and the bifurcations of the OAs. We can clearly separate regimes of FP, FS, BS, CS and ACS (Figs 5C, 5D and 6). As far as we know, an ACS region has never been reported before. We may find narrow regions of chaotic attractors inside the aperiodic regime, although the neuron is not generally chaotic inside the ACS region (see Fig 11C ahead). The aperiodic behavior appears due to failed attempts of spikes (see Fig 7N).

The ISI bifurcation diagrams are shown in Fig 5 (top blue curves). The bottom black points correspond to the amplitude, *A*, of the OAs as function of *T* (top panels) and *x*_*R*_ (bottom panels). The amplitude highlights the presence of SOs, which appear through a supercritical Neimark-Sacker bifurcation of the FP. Except for the transition between SO and ACS, the amplitude continuously varies for all the considered bifurcations in Fig 5; the apparent discontinuity is due to the a fast variation in *A* (within a range of 10^−10^ of *T* or *x*_*R*_). The appearance of large amplitude OA and the qualitative change in oscillations are marked with vertical dotted lines. Fig 5A–5C correspond to approximately the same parameters as those in Fig 4A–4C, respectively.

The FS regime coexists with CS regime in a small range of parameters *T* and *x*_*R*_. Thus, there is a first-order-like transition between these two regimes (see Fig 5A). The transition from BS to FS, on the other hand, happens through increasing duration of a single burst of spikes, Δ*t*_*BS*_, similarly to what happens for the leech heart interneuron modeled by Shilnikov & Cymbalyuk [34] using a conductance-based approach. Fig 5E shows the ISI profile of this transition whereas Fig 6A shows Δ*t*_*BS*_ close to the transition point $x_{R}^{BS}\approx -0.075$ for the same set of parameters. Notice that as the interburst interval (largest ISI) stays nearly steady as $x_{R}\to x_{R}^{BS}$, burst duration increases boundlessly according to $\langle \Delta t_{BS}\rangle ∼{\left|x_{R}-x_{R}^{BS}\right|}^{-1/2}$ characterizing a Homoclinic bifurcation of a Saddle-Node periodic orbit [34].

The border between CS and BS (Fig 5B) is filled up with chaotic attractors due to the loss of stability of cardiac spikes (see e.g. Fig 11A and 11B). In Fig 6C, we show the behavior of the membrane potential, *x*(*t*) versus *t* from Eq (1a), as the transition CS→BS is approached. For small *T* (left plot in Fig 6C), the cardiac spikes are very regular resembling the FitzHugh-Nagumo model [35, 36]. For increasing *T*, the OA loses stability and displays small oscillations even during the slow decrease of the action potential (middle plot). These small oscillations are caused by the subcritial Neimark-Sacker bifurcation of the fast subsystem (Fig 3C), similarly to what happens in early afterdepolarizations of the action potentials of conductance-based models [28, 37]. The BS region starts after a very chaotic region of mixed behavior of cardiac spikes and bursts (right plot) taking place for 0.24 ≲ *T* ≲ 0.25 (the *dust* region in Fig 2A).

The transition ACS→SO happens through an infinite period bifurcation (Fig 5C and 5D). The period of oscillations scales as $\langle ISI\rangle ∼{\left|x_{R}-x_{R}^{CS}\right|}^{-1/2}$ as expected (see Fig 6B) [32, 33]. Here, $x_{R}^{CS}$ is the bifurcation point. The same scaling law holds for 〈*ISI*〉 versus the *T* parameter, around the bifurcation point *T*^*CS*^. The variance of *ISI* diverges close to the transition point $\left(T^{CS};x_{R}^{CS}\right)$ as a power law $\text{Var}\left(ISI\right)∼|x_{R}-x_{R}^{CS}{|}^{-1.8}$ (inset of Fig 6B).

The bifurcation that separates BS and SO (Fig 5B and 5E) is similar to that separating ACS and SO. The largest ISI (or the *interburst interval*, IBI) diverges according to $\langle IBI\rangle ∼{\left|x_{R}-x_{R}^{SO}\right|}^{-1/2}$ and the smaller ISI remains constant. The same power law is found over the *T* parameter.

## 3 Behaviors

This section is dedicated to show that this map exhibits many dynamical behaviors of excitable systems, like neuronal cells and muscle cells, especially the action potential of cardiac cells [38].

**Fig 8:**
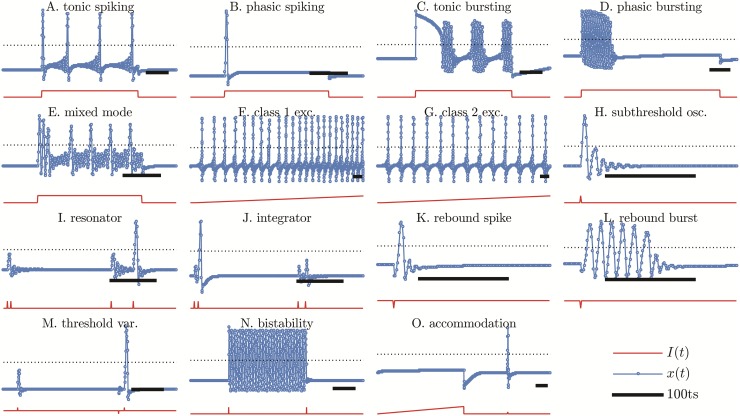
Some excitable behaviors of KTz logistic map. Parameters are given in Table A in S1 File.

In this work, the solution *x*(*t*) of the map, Eq (1a), is the actual membrane potential of an excitable cell. Figs 7 and 8 will have *x* (in arbitrary units) in the vertical axis (plotted with circles and lines) and *t* (in time steps) in the horizontal axis. The solution of Eq (1a) is the set of points plotted in these figures; the lines are there only as a guide to the eyes. Excitable behavior is accompanied by the curve of the input current, *I*(*t*) (in arbitrary units), right below the membrane potential curve. The solution *x*(*t*) corresponds only to circles, but lines are drawn connecting the points in order to guide the eyes of the reader. Parameters for every behavior in both figures are given in Table A in S1 File.

### 3.1 Case I

The bidimensional model can only present three autonomous behaviors: resting state, fast spiking and subthreshold oscillations. Fast spiking in this model is sometimes chaotic. The excitable behaviors in turn are more diverse and are presented in Fig 7A–7F. The excitable behaviors are generally found near the subcritical Neimark-Sacker bifurcation.

The observed excitable behaviors are: relaxation to FP, transient oscillations, tonic spiking, nerve blocking, bistability and excitable spiking. All of them are also present in the original hyperbolic tangent model [10]. However, the amplitude of the logistic model oscillations is somewhat smaller than the original, because the logistic function varies slower for |*T*| → 0 than the hyperbolic tangent.

KT model presents true excitability because, in most bifurcations, a saddle equilibrium separates the basins of attractions of both FPs (see bifurcation diagrams in Section 2). Each curve in Fig 7F is the result of the neuron receiving a small delta pulse stimulus in the first time step of the simulation. The excitability threshold is easily found by simply taking the distance (in the parameter space) between the simulated neuron and the stability limit of the FP calculated in Section 2.

### 3.2 Case II

In addition to the autonomous behaviors of case I, KTz neuron also presents: slow spiking, fast and slow cardiac spiking, aperiodic cardiac spiking, fast and slow bursting and chaotic bursting (mixed cardiac spiking and bursting found in the boundary between these two regimes, as shown in both Figs 7L and 6C center plot).

Cardiac spikes are fully described only by other two differential equation models, namely: the Luo & Rudy model [39] and the FitzHugh-Nagumo model [35, 36]. The first map-based cardiac spike was obtained using the hyperbolic tangent KTz model [8, 11]. The logistic simplification allows us to precisely visualize the mechanism of generating this kind of spikes in maps, differently from other approaches that involve solving the complex differential equations of Luo & Rudy using Euler method with unity step [40].

The existence of the dynamical slow variable *z* generates a large number of slow-fast dynamical behaviors. They are depicted in Fig 8. We quickly explain these behaviors following Izhikevich [21] in order to be able to compare our map-based approach computational efficiency with other models in Section 4.

**Tonic spiking**: Neurons usually present a quiescent behavior until excited by a continuous current, when they start firing spikes periodically (Fig 8A). The train of spikes is present only while stimulated by the external current. This behavior is found in almost every known neuron.

**Phasic spiking**: The neuron fires only once at the beginning of stimulation then becomes quiescent. This is known as phasic spiking (Fig 8B) and is present in general excitable elements.

**Tonic bursting**: KTz neurons are also able to present tonic bursting (Fig 8C) while the continuous current is active. This feature is important since it is related to the production of the gamma oscillations in the brain [21].

**Phasic bursting**: Similarly to phasic spiking, the neuron reacts to the stimuli by a unique burst of spikes (Fig 8D).

**Mixed mode**: Some neurons can exhibit a mixed behavior: they fire a phasic burst spike then start tonic spiking (Fig 8E). Our case II neuron is capable of representing this spiking mode.

**Class 1 excitability**: Some neurons present a variable spiking frequency that depends on the magnitude of the injected current [33]. These neurons are said to display a Class 1 excitability (Fig 8F) and are able to code its input in an unique frequency output. Such behavior may be found in cortical pyramidal neurons and is typically caused by a Saddle-Node on an invariant circle bifurcation [33].

**Class 2 excitability**: There is another class of excitability in which neurons are not capable of firing arbitrarily high frequency spikes (Fig 8G). This behavior is mostly independent of the intensity of the applied current and is typically found in cortical inhibitory interneurons. These spikes are generally caused either by a Saddle-Node bifurcation or by a Neimark-Sacker bifurcation [33].

**Subthreshold oscillations**: The presence of oscillations that are insufficient to trigger a spike is common in a large variety of neurons (Fig 8H). These small oscillations may happen autonomously or after a spike, as pictured in this panel.

**Resonator**: Some neurons can act as resonators. They react only to stimuli in which the frequency is the same as their subthreshold oscillations frequency. In Fig 8I the neuron does not spike when the stimulus frequency is too high.

**Integrator**: In a neuron without subthreshold oscillations, the higher the input frequency, the higher the probability of spiking. This kind of neuron is called integrator (Fig 8J).

**Rebound spike**: Some neurons can fire after an inhibitory input is received. This phenomenon is called post-inhibitory, or rebound, spike (Fig 8K).

**Rebound burst**: Instead of single-peak spike some neurons can fire a burst of spikes after receiving inhibitory stimuli (Fig 8L).

**Threshold variability**: Our model is capable of reproducing the phenomenon of threshold variability (Fig 8M). A stimulus which otherwise would not be able to generate a spike, indeed generates a spike after a small perturbation of the local membrane potential.

**Bistability**: Some neocortical neurons can present two different stable modes. This can be seen in Fig 8N, in which a stable FP and a stable OA coexist. An external stimulus changes the relative positions of the nullclines momentarily and leaves the neuron either in the FP or in the OA basin of attraction.

**Accommodation**: When receiving a slowly increasing continuous input, certain neurons fail to fire. This indicates an accommodation to the stimulus, as shown in Fig 8O. If the same neuron is stimulated by a small and brief input it is able to fire a spike.

### 3.3 Chaotic attractors

**Fig 9:**
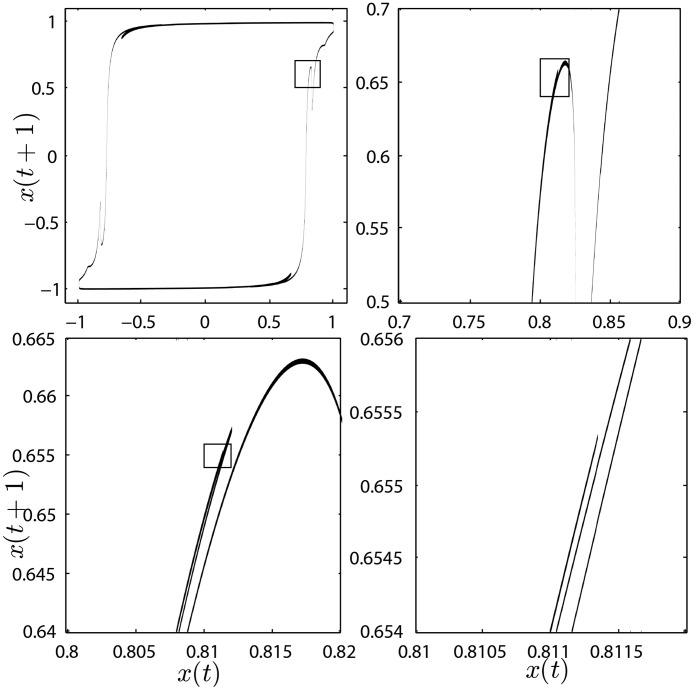
Typical strange attractor for the KTLog map. Parameters *H* = 0, *T* = 0.009, *K* = 0.89 and *x*(0) = *y*(0) = 1. The Lyapunov exponent is *λ*_*L*_ = 0.1258 (see Fig 10). Notice the fractal structure as we zoom in from top-left panel to bottom-right panel.

We use the Eckmann-Ruelle method [41] to calculate the largest Lyapunov exponent, *λ*_*L*_, and find strange attractors. We can find chaotic regions in parameter space for the KTLog model, although these regions are narrower and displaced when compared to the KT model. Typical Lyapunov exponents in the logistic approximation are also smaller than the hyperbolic tangent model. A typical chaotic attractor for the case I and *H* = 0 is in Fig 9. Its capacity dimension is 1.35(2) and the Lyapunov dimension [42] is 1.158(2), thus categorizing it as a strange attractor.

**Fig 10:**
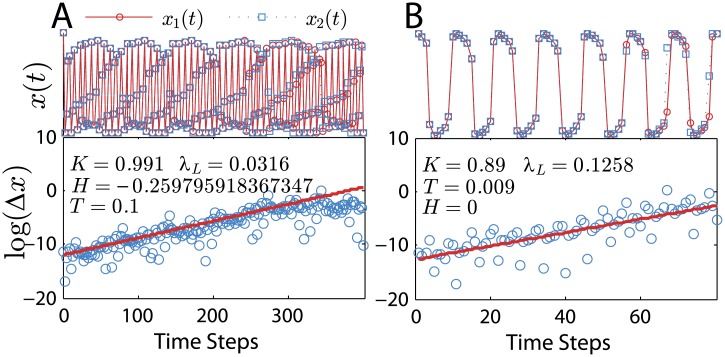
Estimation of Lyapunov exponent via initial condition divergence. Panel A: *λ*_*L*_ = 0.0316 for *H* = −0.259795918367347, *T* = 0.1 and *K* = 0.991. Panel B: *λ*_*L*_ = 0.1258 for *H* = 0, *T* = 0.009 and *K* = 0.89 (same as Fig 9). Initial conditions are *x*(0) = *y*(0) = 1.

We computationally check the chaotic behavior by iterating the map for two sets of initial conditions very close to each other (∼10^−8^) yielding *x*_1_(*t*) and *x*_2_(*t*) and Δ*x*(*t*) = |*x*_2_(*t*)−*x*_1_(*t*)|. Only a few time steps are needed for the separation between the curves to become visible (Fig 10 top panels). We plot Δ*x* versus *t* in Fig 10 bottom panels and estimate the Lyapunov exponent by fitting Δ*x* ∼ exp(*λ*_*L*_ Δ*t*) to the initial divergence of solutions. After some time steps, the curves saturate around Δ*x* ≈ 2 because the solutions *x*_1,2_(*t*) are bounded inside the range [−1; 1].

Eckmann-Ruelle method applied to both cases of Fig 10 gives *λ*_*L*_ ≈ 0.027 for panel A and *λ*_*L*_ ≈ 0.122 for panel B. These values are very close to the exponentially fitted exponents. Both regimes correspond to case I with initial conditions *x*(0) = *y*(0) = 1.

**Fig 11:**
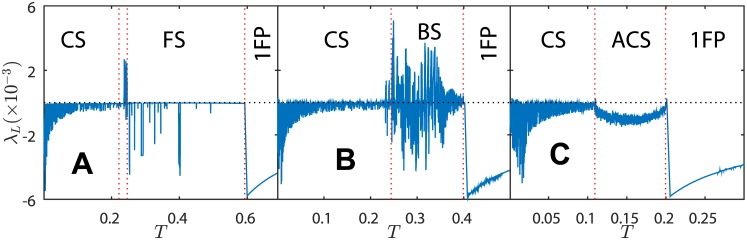
Lyapunov exponent of the KTzLog map. Parameters *K* = 0.6 and *δ* = *λ* = 0.001; *T* is the control parameter. Panel A: *x*_*R*_ = −0.01; Panel B: *x*_*R*_ = −0.2; Panel C: *x*_*R*_ = −0.4. These parameters are the same as in Fig 5A–5C, respectively, and correspond to the horizontal dotted lines in Fig 2A.

We also applied this method to determine the largest Lyapunov exponent for a vast region of parameters of case II. We show *λ*_*L*_ as function of *T* in Fig 11A–11C for the same set of parameters of Fig 5A–5C, respectively.

In fact, Fig 11 shows the Lyapunov exponent over the horizontal dotted lines of the *x*_*R*_ × *T* diagram in Fig 2. Notice that chaotic behavior is very strong on the frontier between CS and BS and inside BS region. Also, positive Lyapunov exponent has been found on the edges of ACS region (Fig 11C).

## 4 Computational efficiency

Usually, the performance of a program depends on many uncontrollable variables, such as code style, language specific implementations, background memory operations and usage, function calls, concurrent processing due to operating system threads, etc. Thus, there is a diversity of measures for computational efficiency. Izhikevich [21] defines it through the amount of *floating-point operations* (FLOPs) the neuron model needs in order to evolve 1 ms (model time) of its dynamics, considering that a spike takes around 1 ms to rise and fall.

The intrinsic dynamics of the model also matters for performance: after a spike, each model reaches the FP after different number of time steps. For instance, the KTzLog model takes only 136 ts (roughly 1,632 FLOPs) to reach the FP whereas the Izhikevich model takes 1,158 ts (roughly 15,054 FLOPs). These convergence times clearly depend on the specific phase portrait of the model for the chosen parameters. Thus, we follow a similar approach to Girardi-Schappo *et al*. [8] and measure the CPU cycles needed for a single time step of the model to be evaluated as a better measure of efficiency. The comparison of these and other efficiency-related measures (FLOPs and convergence to FP) are given in Table 1 for the more popular models. A CPU cycle is the fundamental operation cycle of a computer and thus it takes into consideration all the processing operations. It consists of retrieving a program instruction from the memory, determining the needed actions to process the instruction and executing these actions [43].

**Table 1:** Computational efficiency of some standard single-neuron models. The *ts to FP* column is the number of time steps taken to approach the FP with a tolerance of 10^−8^. The *ms/ts* column shows the proposed scale factors for the time step of each model, assuming a spikes takes about 1 ms. The number of FLOPs for the conductance based model is estimated assuming a 4*^th^* order Runge-Kutta ODE solver with step 0.001 ms. Each model’s average CPU cycles has been performed over 5,000 realizations of 3,000 ms considering the lowest conversion factor in the table for the considered model.

Model	FLOPs/ts	ts to FP	ms/ts	CPU cycles/ts
**Rulkov** [13]	14	561	0.5	37 ± 7
**Izhikevich** [14]	13	1,158	0.5 to 1	36 ± 8
**KTzLog**	12	136	0.1 to 0.2	60 ± 13
**Tanh KTz** [11]	31	554	0.1 to 0.2	99 ± 18
**Conductance model** [34]	124	9,763	0.001	795 ± 32

**Fig 12:**
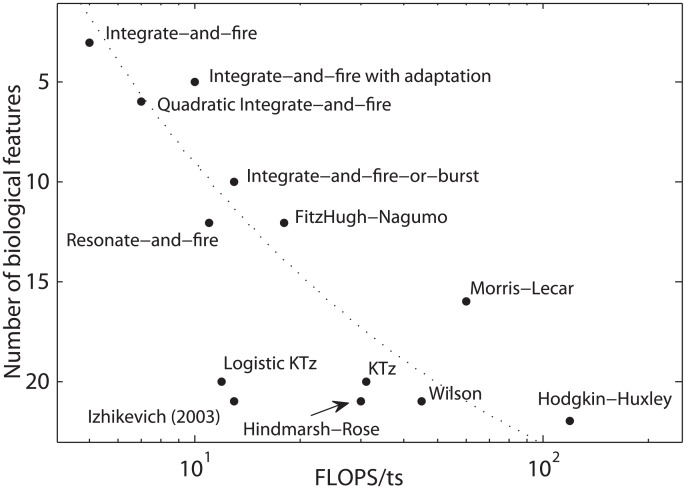
Neuronal models efficiency diagram. Scheme of the number of *biological features* displayed by a model versus the amount of floating-point operations (FLOPS) needed for each time step of the model. Adapted from Izhikevich [21] to include logistic and hyperbolic KTz models.

The number of FLOPs/ts of both logistic and hyperbolic KTz models are 12 FLOPs/ts and 31 FLOPs/ts, respectively. We show them in Fig 12 together with other standard models and their quantity of biological features. We estimated the quantity of biological features of our neuron model through Figs 7 and 8. Izhikevich [21] defines a set of 22 biological features in order to measure “biological relevance” of the model. However, notice that some features are not accounted for in the proposed list, such as cardiac spikes, nerve blocking and transient oscillations. Out of that list, we found 15 biological features in both logistic and hyperbolic KTz plus these three aforementioned behaviors. It does not mean that the remaining 7 features of Izhikevich’s list are not present in KTz family, because there is a lot more to explore in our proposed neuron map.

Map-based models are seven to twenty times more efficient than ordinary differential equation (ODE) models if we consider the CPU cycles in Table 1. Even though Rulkov, Izhikevich and our model have similar number of FLOPs/ts, our model time step takes about one and a half times more CPU cycles to be evaluated than the other two models. These extra cycles are needed in order to deal with possible overflow operations for large absolute arguments of the logistic function during map evaluation. Notice however that the logistic approximation optimized both the FP convergence time (in ts) and the quantity of CPU cycles per time step in relation to the hyperbolic model. And, more interestingly, the logistic approximation kept all the dynamical behaviors of the hyperbolic KTz.

We also consider two typical network cases: linear and mean-field. In both cases, there are *N* neurons and the synaptic current over an element *i* is given by a gap junction model [8]:

$$\begin{array}{c} I_{i}^{syn}\left(t\right)=\sum_{j}G\left[x_{j}\left(t\right)-x_{i}\left(t\right)\right], \end{array}$$ (21)

where the sum runs over the neighbors of *i* and *G* > 0 is an excitatory conductance that is adjusted for each case slightly above the necessary threshold to propagate activity. The coupling scheme for each map-based model is described in details elsewhere [8]. The linear network is directional (with a total of *N* − 1 synapses), neurons are adjusted in an excitable regime and only the left-most neuron is initially excited (*i* = 1). The signal propagates until it eventually reaches neuron *i* = *N*. The mean-field network is a complete graph without self-interactions [total of *N*(*N* − 1) synapses], and random initial conditions, so the neurons (adjusted in a bursting regime) tend to synchronize after some transient activity. The conductance-based model is either the standard Hodgkin-Huxley model for the linear network or the leech heart interneuron model given by [34] due to its bursting regime.

**Fig 13:**
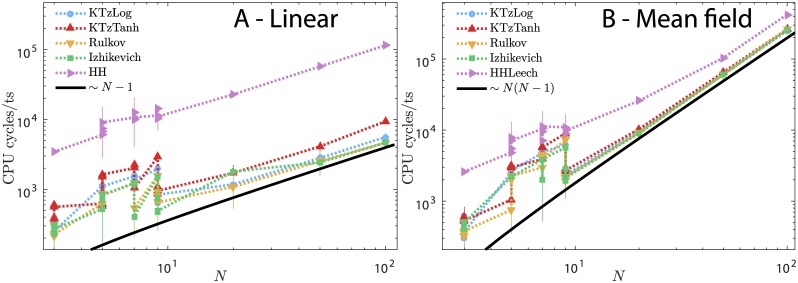
Network efficiency comparison. CPU cycles per time step for networks of *N* neurons using each of the considered models as the base element. Panel A: linear network (directional synapses from 1 to *N*). Panel B: complete graph (each neuron connects to all the other neurons, and *vice-versa*). Synapses are modeled by gap junctions.

Fig 13 shows the CPU cycles/ts for each network of each model. Here, the network time step is evaluated as follows: 1) calculate the signal of every synapse; 2) for each neuron, sum up its input signal; 3) calculate the new membrane potential of each neuron considering its input current.

Both networks have similar trends as *N* is increased. The number of CPU cycles/ts increases proportionally to the number of synapses in the network for large *N* (notice the black solid curve in both panels of Fig 13). The conductance-based models demand significantly more computational power. Except for the tanh KTz, the KTzLog, Izhikevich and Rulkov maps have equivalent efficiency. The large fluctuations for small *N* appear because the background operating system processes have a high impact on the CPU cycles of fast jobs. It is worth noticing that the precise number of CPU cycles strongly depends on the processor, memory architecture and operating system used to compile and run the program. However, the statistical trend should still hold for different computers. We used Microsoft(R) Windows(TM) 10, version 10.0.14393 Build 14393, on a x64 Intel(R) Core(TM) i7-4500U CPU @ 1.80GHz, 2401 Mhz, 2 Cores (4 Logical Processors), using 16 GB of DDR3-RAM.

## 5 Concluding remarks

We studied a model of action potential generation using difference equations obtained through the first order Taylor approximation of the hyperbolic tangent KTz neuron model [11]. We presented detailed bifurcation diagrams and fixed point stability diagrams for our new model, the so-called KTzLog neuron. We have shown that the KTzLog neuron reproduces many neuronal excitatory and autonomous behaviors observed experimentally. We have compared our model’s computational performance to other widely used models and concluded that its efficiency is comparable to that of the most efficient neuron models, both in isolation or in a network.

We highlight the presence of cardiac spikes within a large parameter region of the KTzLog model. This behavior is only possible because we do not follow an *integrate-and-fire* procedure for spike generation. Instead, the action potential of our model has its own slow-fast dynamics. Other authors have attempted cardiac spikes modeling, either by discretizing Luo & Rudy model [40], or by proposing piecewise-continuous functions [44]. Or yet, some authors proposed the modeling of the action potential duration through a difference equation [45]. However, only the KTz map family displays cardiac spikes with a reduced set of parameters on top of a simple continuous sigmoid dynamics. This system was fundamentally conceived as a map [8] and the logistic approximation allows us to analytically study bifurcations and stability of orbits without loss of any dynamical behavior. This fact may allow us to better understand phenomena such as early afterdepolarization and cardiac arrhythmia: we have identified a bifurcation that is known to cause early afterdepolarized action potentials in ODE neurons [37].

An aperiodic cardiac spike phase was found in a large parameter region of both versions of KTz model (logistic and tanh). Surprisingly, the Lyapunov exponents inside this phase are negative, indicating a non-chaotic behavior. We are not aware of another work that has shown the existence of this non-chaotic and aperiodic behavior in excitable systems. We will investigate these subjects in forthcoming works.

Mesbah *et al*. [16] have proposed a uni-dimensional map-based neuron model that displays a few excitable behaviors. However, such behaviors are obtained by applying external currents to different parameters, making it very hard for neurons to be coupled in order to produce an heterogeneous neuronal network (as the brain is expected to be). On the other hand, external input currents and synaptic coupling is easily attainable by adding a current term to the KTzLog model equation similarly to what is done with conductance based models. An example of synaptic map was proposed by Kuva *et al*. [11], and used by Girardi-Schappo *et al*. [22]to study neuronal avalanches. Thus, it is straightforward to build biologically motivated networks of KTz maps in order to study higher brain functions, such as cortical processing, synchronization phenomena and so on.

KTzLog map has only five parameters (or six, if *H* is considered a polarizing current for the three-dimensional model) displaying almost as many excitable behaviors as the Izhikevich [14] model (which has nine parameters). In this sense, the KTz family, and specially its logistic variation, provides minimal models to study neuronal bifurcations. Also, the logistic case displays all of its bifurcations in their normal forms, making the KTzLog model one of the canonical neuron models of choice for studying unknown mechanisms underlying dynamical phenomena in neurons (such as early afterdepolarization).

Modeling complex functions of the nervous system demands an extensive gathering of information about the system one wants to mimic. During this process, a certain degree of simplification is unavoidable. Neurons are the basic processing units of the brain, but they are generally the first elements of the simulation that are reduced. However, these simplifications are sometimes useful, as they may result in a more reliable model in the context of the function one is trying to understand [6, 46]. On the other hand, modelers must use models that allow a certain degree of controllability of its neurons’ dynamics in order to be able to make inferences about the simulated experimental setup. In any case, the chosen neuron model should be capable to provide simultaneously a good computational performance and enough features to make the model reliable and easy to use. We have shown that the KTzLog model meet these essential requirements.

Furthermore, future work with our model include understanding the chaotic attractors and their coexistence with periodic attractors, the shrimp structure in the *x*_*R*_ × *T* diagram, compartmental modeling, cardiac spike bifurcations and cardiac tissue modeling.

## Supporting information

S1 FileSupporting information: Phase diagrams and dynamics of a computationally efficient map-based neuron model.Details about the ISI method used to determine the OA in this paper and about the parameters of the model for each behavior depicted in Figs 7 and 8. **Fig A, Typical ISI distributions**. The four different types of ISI distribution P(ISI) are displayed in panels A to D (top) with their corresponding map iteration (bottom). Solid and dashed lines are only there to guide the eyes. Panel A: Fast spiking (FS)—a single well defined peak in P(ISI) such that 〈*ISI*〉 < *ISI*_*th*_. Panel B: Bursting (BS)—two peaks are generally present in P(ISI) for BS behavior; if chaotic bursting is present, both peaks will be broadened; slow bursting phase has the large ISI larger than an arbitrary threshold. Panel C: Periodic cardiac spiking (CS)—a single peak in P(ISI) such that 〈*ISI*〉 > *ISI*_*th*_. Panel D: Aperiodic cardiac spiking (ACS)—a single peak broad distribution P(ISI) shaped similarly to a lognormal curve. Fixed point (FP) has no ISI. **Table A, Parameters for the reproduction of all KTzLog behaviors in Figs 7 and 8 of the main text**.(PDF)

## References

[1] Izhikevich EM, Edelman GM. Large-scale model of mammalian thalamocortical systems. Proc Natl Acad Sci USA. 2008;105(9):3593–3598. 10.1073/pnas.0712231105 18292226 PMC2265160

[2] de Garis H, Shuo C, Goertzel B, Ruiting L. A world survey of artificial brain projects, Part I: Large-scale brain simulations. Neurocomputing. 2010;74:3–29. 10.1016/j.neucom.2010.08.004

[3] Eliasmith C, Stewart TC, Choo X, Bekolay T, DeWolf T, Tang Y, et al A Large-Scale Model of the Functioning Brain. Science. 2012;338:1202–1205. 10.1126/science.1225266 23197532

[4] Potjans TC, Diesmann M. The Cell-Type Specific Cortical Microcircuit: Relating Structure and Activity in a Full-Scale Spiking Network Model. Cereb Cortex. 2014;24(3):785–806. 10.1093/cercor/bhs358 23203991 PMC3920768

[5] Markram H, Muller E, Ramaswamy S, Reimann MW, Abdellah M, Sanchez CA, et al Reconstruction and Simulation of Neocortical Microcircuitry. Cell. 2015;163(2):456–492. 10.1016/j.cell.2015.09.029 26451489

[6] Herz AVM, Gollisch T, Machens CK, Jaeger D. Modeling Single-Neuron Dynamics and Computations: A Balance of Detail and Abstraction. Science. 2006;314:80–85. 10.1126/science.1127240 17023649

[7] Ibarz B, Casado JM, Sanjuán MAF. Map-based models in neuronal dynamics. Phys Rep. 2011;501:1–74. 10.1016/j.physrep.2010.12.003

[8] Girardi-Schappo M, Kinouchi O, Tragtenberg MHR. A brief history of excitable map-based neurons and neural networks. J Neurosci Methods. 2013;220(2):116–130. 10.1016/j.jneumeth.2013.07.014 23916623

[9] Chialvo DR. Generic Excitable Dynamics on a Two-dimensional Map. Chaos Solitons Fractals. 1995;5:461–479. 10.1016/0960-0779(93)E0056-H

[10] Kinouchi O, Tragtenberg MHR. Modeling neurons by simple maps. Int J Bifurcat Chaos. 1996;6:2343–2360. 10.1142/S0218127496001508

[11] Kuva SM, Lima GF, Kinouchi O, Tragtenberg MHR, Roque AC. A minimal model for excitable and bursting elements. Neurocomputing. 2001;38–40:255–261. 10.1016/S0925-2312(01)00376-9

[12] Rulkov NF. Regularization of synchronized chaotic bursts. Phys Rev Lett. 2001;86:183–186. 10.1103/PhysRevLett.86.183 11136124

[13] Rulkov NF. Modeling of spiking-bursting neural behavior using two-dimensional map. Phys Rev E. 2002;65:041922 10.1103/PhysRevE.65.041922 12005888

[14] Izhikevich EM, Hoppensteadt F. Classification of Bursting Mappings. Int J Bifurcat Chaos. 2004;14(11):3847–3854. 10.1142/S0218127404011739

[15] Courbage M, Nekorkin VI, Vdovin LV. Chaotic oscillations in a map-based model of neural activity. Chaos. 2007;17(4):043109 10.1063/1.2795435 18163773

[16] Mesbah S, Moghtadaei M, Golpayegani MRH, Towhidkhah F. One-dimensional map-based neuron model: A logistic modification. Chaos Solitons Fractals. 2014;65:20–29. 10.1016/j.chaos.2014.04.006

[17] Weiss JN, Garfinkel A, Karagueuzian HS, Sheng Chen P, Qu Z. Early afterdepolarizations and cardiac arrhythmias. Heart Rhythm. 2010;7(12):1891–1899. 10.1016/j.hrthm.2010.09.017 20868774 PMC3005298

[18] Quail T, Shrier A, Glass L. Predicting the onset of period-doubling bifurcations in noisy cardiac systems. Proc Nat Acad Sci (USA). 2015;112(30):9358–9363. 10.1073/pnas.1424320112 26170301 PMC4522826

[19] McCulloch WS, Pitts WH. A Logical Calculus of the Ideas Immanent in Nervous Activity. Bull Math Biophys. 1943;5:115–133. 10.1007/BF02478259 2185863

[20] Tragtenberg MHR, Yokoi CSO. Field behavior of an Ising model with competing interactions on the Bethe lattice. Phys Rev E. 1995;52(3):2187–2197. 10.1103/PhysRevE.52.2187 9963658

[21] Izhikevich EM. Which model to use for cortical spiking neurons? IEEE Trans Neural Netw. 2004;15:1063–1070. 10.1109/TNN.2004.832719 15484883

[22] Girardi-Schappo M, Kinouchi O, Tragtenberg MHR. Critical avalanches and subsampling in map-based neural networks coupled with noisy synapses. Phys Rev E. 2013;88:024701 10.1103/PhysRevE.88.024701 24032969

[23] Amir R, Michaelis M, Devor M. Burst Discharge in Primary Sensory Neurons: Triggered by Subthreshold Oscillations, Maintained by Depolarizing Afterpotentials. J Neurosci. 2002;22(3):1187–1198. 11826148 10.1523/JNEUROSCI.22-03-01187.2002 PMC6758504

[24] Tao Zhu Z, Munhall A, Zhong Shen K, Johnson SW. Calcium-dependent subthreshold oscillations determine bursting activity induced by N-methyl-d-aspartate in rat subthalamic neurons in vitro. Eur J Neurosci. 2004;19:1296–1304. 10.1111/j.1460-9568.2004.03240.x 15016087

[25] Sato D, Hua Xie L, Sovari AA, Tran DX, Morita N, Xie F, et al Synchronization of chaotic early afterdepolarizations in the genesis of cardiac arrhythmias. Proc Nat Acad Sci (USA). 2009;106(9):2983–2988. 10.1073/pnas.0809148106 19218447 PMC2651322

[26] White JA, Budde T, Kay AR. A Bifurcation Analysis of Neuronal Subthreshold Oscillations. Biophys J. 1995;69:1203–1217. 10.1016/S0006-3495(95)79995-7 8534792 PMC1236352

[27] Izhikevich EM. Resonance and selective communication via bursts in neurons having subthreshold oscillations. BioSystems. 2002;67:95–102. 10.1016/S0303-2647(02)00067-9 12459288

[28] Tran DX, Sato D, Yochelis A, Weiss JN, Garfinkel A, Qu Z. Bifurcation and Chaos in a Model of Cardiac Early Afterdepolarizations. Phys Rev Lett. 2009;102:258103 10.1103/PhysRevLett.102.258103 19659123 PMC2726623

[29] Copelli M, Tragtenberg MHR, Kinouchi O. Stability diagrams for bursting neurons modeled by three-variable maps. Physica A. 2004;342:263–269. 10.1016/j.physa.2004.04.087

[30] Gallas JAC. Dissecting shrimps: results for some one-dimensional physical models. Physica A. 1994;202:196–223. 10.1016/0378-4371(94)90174-0

[31] de Souza SLT, Batista AM, Baptista MS, Caldas IL, Balthazar JM. Characterization in bi-parameter space of a non-ideal oscillator. Phys A Stat Mech its Appl. 2017;466:224–231. 10.1016/j.physa.2016.09.020

[32] Strogatz SH. Nonlinear Dynamics and Chaos: With Applications to Physics, Biology, Chemistry, and Engineering. Westview Press; 2001.

[33] Izhikevich EM. Dynamical Systems in Neuroscience. Cambridge, Massachussetts, USA: The MIT Press; 2007.

[34] Shilnikov A, Cymbalyuk G. Transition between Tonic Spiking and Bursting in a Neuron Model via the Blue-Sky Catastrophe. Phys Rev Lett. 2005;94:048101 10.1103/PhysRevLett.94.048101 15783604

[35] FitzHugh R. Mathematical models of threshold phenomena in the nerve membrane. Bulletin of Mathematical Biophysics. 1955;17:257–278. 10.1007/BF02477753

[36] Nagumo J, Arimoto S, Yoshizawa S. An active pulse transmission line simulating nerve axon. Proceedings of the IRE. 1962;50:2061–2070. 10.1109/JRPROC.1962.288235

[37] Kügler P. Early Afterdepolarizations with Growing Amplitudes via Delayed Subcritical Hopf Bifurcations and Unstable Manifolds of Saddle Foci in Cardiac Action Potential Dynamics. PLoS ONE. 2016;11(3):e0151178 10.1371/journal.pone.0151178 26977805 PMC4792449

[38] Fenton FH, Cherry EM. Models of cardiac cell. Scholarpedia. 2008;3(8):1868 10.4249/scholarpedia.1868

[39] Luo CH, Rudy Y. A model of the ventricular cardiac action potential. Depolarization, repolarization, and their interaction. Circ Res. 1991;68(6):1501–1526. 10.1161/01.RES.68.6.1501 1709839

[40] Pavlov EA, Osipov GV, Chan CK, Suykens JAK. Map-based model of the cardiac action potential. Phys Lett A. 2011;375:2894–2902. 10.1016/j.physleta.2011.06.014

[41] Eckmann JPP, Ruelle D. Ergodic theory of chaos and strange attractors. Rev Mod Phys. 1985;57(3):617–656. 10.1103/RevModPhys.57.617

[42] Frederickson P, Kaplan JL, Yorke ED, Yorke JA. The Liapunov Dimension of Strange Attractors. J Differ Equ. 1983;49(2):185–207. 10.1016/0022-0396(83)90011-6

[43] Hennessy JL, Patterson DA. Computer Architecture: A quantitative approach. Waltham, MA, USA: Morgan Kaufmann; 2012.

[44] Rulkov NF. A Map-Based Model of the Cardiac Action Potential. arXiv:07081173v1 [q-bioCB]. 2007;.

[45] Tolkacheva EG, Schaeffer DG, Gauthier DJ, Mitchell CC. Analysis of the Fenton–Karma model through an approximation by a one-dimensional map. Chaos. 2002;12(4):1034–1042. 10.1063/1.1515170 12779627

[46] Brette R. What Is the Most Realistic Single-Compartment Model of Spike Initiation? PLoS Comput Biol. 2015;11(4):e1004114 10.1371/journal.pcbi.1004114 25856629 PMC4391789

