# Supplementary material for: Phase diagrams and dynamics of a computationally efficient map-based neuron model
Source: PLoS One. 2017 Mar 30;12(3):e0174621. doi: 10.1371/journal.pone.0174621 (PMC5373601; doi:10.1371/journal.pone.0174621)
Supplement: S1 File — Details about the ISI method used to determine the OA in this paper and about the parameters of the model for each behavior depicted in Figs 7 and 8. Fig A, Typical ISI distributions. The four different types of ISI distribution P(ISI) are displayed in panels A to D (top) with their corresponding map iteration (bottom). Solid and dashed lines are only there to guide the eyes. Panel A: Fast spiking (FS)—a single well defined peak in P(ISI) such that 〈ISI〉 < ISIth. Panel B: Bursting (BS)—two peaks are generally present in P(ISI) for BS behavior; if chaotic bursting is present, both peaks will be broadened; slow bursting phase has the large ISI larger than an arbitrary threshold. Panel C: Periodic cardiac spiking (CS)—a single peak in P(ISI) such that 〈ISI〉 > ISIth. Panel D: Aperiodic cardiac spiking (ACS)—a single peak broad distribution P(ISI) shaped similarly to a lognormal curve. Fixed point (FP) has no ISI. Table A, Parameters for the reproduction of all KTzLog behaviors in Figs 7 and 8 of the main text. (PDF) [file pone.0174621.s001.pdf]

Supplementary information: Phase Diagrams and Dynamics of a Computationally Efficient Map-based Neuron Model

Mauricio Girardi-Schappo[1], Germano S. Bortolotto[2], Rafael V. Stenzinger[2], Jheniffer J. Gonsalves[2], Marcelo H. R. Tragtenberg[2*],

1 Neuroimaging of Epilepsy Laboratory, McConnell Brain Imaging Center, McGill University, Montreal Neurological Institute and Hospital, H3A 2B4, Montreal, Quebec, Canada
2 Departamento de Física, Universidade Federal de Santa Catarina, 88040-900, Florianópolis, Santa Catarina, Brazil

* marcelotragtenberg@gmail.com

Abstract

We introduce a new map-based neuron model derived from the dynamical perceptron family that has the best compromise between computational efficiency, analytical tractability, reduced parameter space and many dynamical behaviors. We calculate bifurcation and phase diagrams analytically and computationally that underpins a rich repertoire of autonomous and excitable dynamical behaviors. We report the existence of a new regime of cardiac spikes corresponding to nonchaotic aperiodic behavior. We compare the features of our model to standard neuron models currently available in the literature.

1 Interspike interval

We also calculate the *interspike interval* (ISI) to be able to separate different oscillatory behavior both in bifurcation diagrams and in phase diagrams. The bifurcation of ISI is a standard method in the literature both *in vitro* and in simulations to characterize individual processing of information (see, e.g., Refs. [1–5]). We define the ISI, measured in ts, as the time interval between every consecutive spike in the map iteration time series. A spike happens at instant $t_{sp}^{(j)}$ if $x(t_{sp}^{(j)}+1)x(t_{sp}^{(j)})<0$, with $x(t_{sp}^{(j)}+1)<x(t_{sp}^{(j)})$, so that $ISI_j=t_{sp}^{(j+1)}-t_{sp}^{(j)}$. The ISI_j are distributed according to $\mathcal{P}(ISI)$ with average $\langle ISI\rangle$. For examples of ISI distributions, see Fig. A. We plot all ISI_j versus T and x_R as bifurcation diagrams in Fig. 5.

We define a threshold, $ISI_{th}=20$ ts in order to separate oscillatory phases as follows: FS has one small characteristic $ISI_j=\langle ISI\rangle<ISI_{th}$; CS regime has one characteristic $ISI_j=\langle ISI\rangle>ISI_{th}$; and bursting spiking (BS) is characterized by having both $ISI_j>ISI_{th}$ and $ISI_j<ISI_{th}$. FP has no ISI. An *aperiodic cardiac spike* (ACS) region has been identified. It is characterized by having infinitely many ISI_j all distributed around a well defined average $\langle ISI\rangle>ISI_{th}$ (see Fig. 5C inset).

2 Simulation parameters

The parameters for each panel in Figs. 7 and 8 of the main text are given in Table A. We used standard stimulation procedure to obtain the described excitatory behavior:

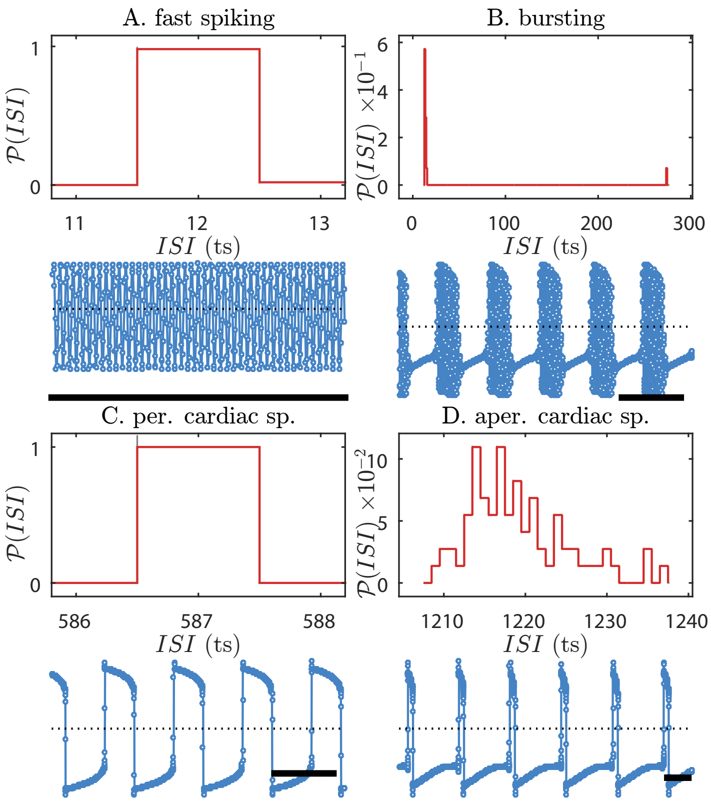

Figure A. Typical ISI distributions. The four different types of ISI distribution $\mathcal{P}(ISI)$ are displayed in panels A to D (top) with their corresponding map iteration (bottom). Solid and dashed lines are only there to guide the eyes. Panel A: Fast spiking (FS) – a single well defined peak in $\mathcal{P}(ISI)$ such that $\langle ISI \rangle < ISI_{th}$. Panel B: Bursting (BS) – two peaks are generally present in $\mathcal{P}(ISI)$ for BS behavior; if chaotic bursting is present, both peaks will be broadened; slow bursting phase has the large ISI larger than an arbitrary threshold. Panel C: Periodic cardiac spiking (CS) – a single peak in $\mathcal{P}(ISI)$ such that $\langle ISI \rangle > ISI_{th}$. Panel D: Aperiodic cardiac spiking (ACS) – a single peak broad distribution $\mathcal{P}(ISI)$ shaped similarly to a lognormal curve. Fixed point (FP) has no ISI.

current pulses $I(t) = I_0\delta_{t,t_0}$, current steps $I(t) = I_0\left[\Theta\left(t - t_0\right) - \Theta\left(t - t_1\right)\right]$ and current ramps $I(t) = a(t - t_0)$, where δ_{t,t_0} is the Kronecker delta and $\Theta(t - t_0)$ is the Heaviside step function.

References

1. Reich DS, Mechler F, Purpura KP, Victor JD. Interspike Intervals, Receptive Fields, and Information Encoding in Primary Visual Cortex. J Neurosci. 2000;20(5):1964–1974.

2. Wu XB, Mo J, Yang MH, Zheng QH, Gu HG, Ren W. Two Different Bifurcation Scenarios in Neural Firing Rhythms Discovered in Biological Experiments by Adjusting Two Parameters. Chin Phys Lett. 2008;25(8):2799–2802. doi:10.1088/0256-307X/25/8/018.

Table A. Parameters for the reproduction of all KTzLog behaviors in Figs. 7 and 8 of the main text.

Behavior	Fig.	K	T	δ	λ	x_R	H	$\vec{x}(0)$	Input current
**fixed point**	7A	0.6	0.35	0	0	0	-0.06	$(-0.375, -0.375, 0)$	Step:$I_0 = 0.04$; $t_0 = 150$ ts; $t_1 = 350$ ts
**transient osc.**	7B	0.6	0.35	0	0	0	-0.06	$(-0.375, -0.375, 0)$	Step:$I_0 = 0.04185$; $t_0 = 150$ ts; $t_1 = 350$ ts
**tonic spiking**	7C	0.6	0.35	0	0	0	-0.06	$(-0.375, -0.375, 0)$	Step:$I_0 = 0.05$; $t_0 = 150$ ts; $t_1 = 350$ ts
**nerve blocking**	7D	0.6	0.35	0	0	0	-0.06	$(-0.375, -0.375, 0)$	Ramp: $a = 0.0005$ ts^{-1}; $t_0 = 100$ ts
**bistablity**	7E	0.6	0.35	0	0	0	-0.014	$(-0.237, -0.237, 0)$	Two pulses: $I_0 = 0.1$, $t_0 = 150$ ts; $I_1 = -0.09$, $t_1 = 312$ ts
**excitability**	7F	0.6	0.3	0	0	0	-0.05	$(-0.422, -0.422, 0)$	Many pulses with $I_0 \leq 0.13$, $t_0 = 10$ ts
**fast spiking**	7G	0.6	0.3	0.001	0.001	-0.05	0	$(0, 0, 0)$	-
**slow spiking**	7H	0.6	0.21	0.01	0.01	-0.37	0	$(0, 0, 0)$	-
**cardiac spiking**	7I	0.6	0.2	0.001	0.001	-0.2	0	$(0, 0, 0)$	-
**fast bursting**	7J	0.6	0.27	0.001	0.001	-0.2	0	$(0, 0, 0)$	-
**slow bursting**	7K	0.6	0.27	0.001	0.001	-0.3	0	$(0, 0, 0)$	-
**chaotic bursting**	7L	0.6	0.25	0.001	0.001	-0.15	0	$(0, 0, 0)$	-
**subthreshold osc.**	7M	0.6	0.23	0.001	0.008	-0.38	0	$(-0.39, -0.39, 0.00891)$	-
**aperiodic CS**	7N	0.6	0.2	0.001	0.001	-0.4	0	$(-0.423, -0.423, 0.0229)$	-
**tonic spiking**	8A	0.6	0.19	0.051	0.015	-0.59	0	$(-0.549, -0.549, -0.0119)$	Step:$I_0 = 0.069$; $t_0 = 200$ ts; $t_1 = 700$ ts
**phasic spiking**	8B	0.6	0.15	0.05	0.03	-0.7	0	$(-0.658, -0.658, -0.0253)$	Step:$I_0 = 0.1$; $t_0 = 100$ ts; $t_1 = 400$ ts
**tonic bursting**	8C	0.6	0.3	0.001	0.003	-0.3	0	$(-0.297, -0.297, -0.00801)$	Step:$I_0 = 0.3$; $t_0 = 200$ ts; $t_1 = 700$ ts
**phasic bursting**	8D	0.6	0.3	0.001	0.001	-0.35	0	$(-0.333, -0.333, -0.0167)$	Step:$I_0 = 0.04$; $t_0 = 100$ ts; $t_1 = 900$ ts
**mixed mode**	8E	0.6	0.3	0.04	0.01	-0.59	0	$(-0.411, -0.411, -0.0448)$	Step:$I_0 = 0.07$; $t_0 = 100$ ts; $t_1 = 400$ ts
**class 1 exc.**	8F	0.6	0.23	0.001	0.008	-0.38	0	$(-0.39, -0.39, 0.00891)$	Ramp: $a = 0.0001$ ts^{-1}; $t_0 = 100$ ts
**class 2 exc.**	8G	0.6	0.23	0	0.008	-0.38	0	$(-0.39, -0.39, 0.00891)$	Ramp: $a = 0.0001$ ts^{-1}; $t_0 = 100$ ts
**subthreshold osc.**	8H	0.6	0.25	0.1	0.0058	-0.52	0	$(-0.401, -0.401, -0.00691)$	Pulse: $I_0 = 0.1$; $t_0 = 20$ ts
**resonator**	8I	0.6	0.25	0.04	0.01	-0.4	0	$(-0.387, -0.387, -0.00314)$	Four pulses: $I_0 = 0.05$; $\Delta t_1 = 8$ ts; $\Delta t_2 = 51$ ts
**integrator**	8J	0.6	0.2	0.04	0.05	-0.5	0	$(-0.5, -0.5, 0)$	Four pulses: $I_0 = 0.07$; $\Delta t_1 = 9$ ts; $\Delta t_2 = 17$ ts
**rebound spike**	8K	0.6	0.3	0.001	0.008	-0.35	0	$(-0.347, -0.347, -0.0207)$	Pulse: $I_0 = -0.2$; $t_0 = 20$ ts
**rebound burst**	8L	0.6	0.3	0.004	0.001	-0.35	0	$(-0.308, -0.308, -0.0104)$	Pulse: $I_0 = -0.09$; $t_0 = 20$ ts
**threshold var.**	8M	0.6	0.15	0.06	0.01	-0.5	0	$(-0.598, -0.598, 0.0163)$	Three pulses: $I_0 = I_2 = 0.1$; $I_1 = -0.1$; $t_0 = 50$ ts; $t_1 = 400$ ts; $t_2 = 420$ ts
**bistability**	8N	0.6	0.23	0	0	-0.1	0	$(-0.425, -0.425, 0)$	Two pulses: $I_0 = 0.2$; $t_0 = 200$ ts; $t_1 = 605$ ts
**accommodation**	8O	0.6	0.3	0.001	0.008	-0.35	0	$(-0.347, -0.347, -0.0207)$	Two ramps: $a_0 = 0.0002$ ts^{-1}, $t_0 = 0$ ts; and $a_1 = 0.005$ ts^{-1}, $t_1 = 2500$

3. Ostojic S. Interspike interval distributions of spiking neurons driven by fluctuating inputs. J Neurophysiol. 2011;106:361–373. doi:10.1152/jn.00830.2010.

4. Gu H, Pan B, Xu J. Bifurcation Scenarios of Neural Firing Patterns across Two Separated Chaotic Regions as Indicated by Theoretical and Biological Experimental Models. Abstract and Applied Analysis. 2013;2013:374674. doi:10.1155/2013/374674.

5. Wang L, Liu S. The Effects of Leakage Conductance on Firing Properties in a Compartment Neuron Model. In: Yamaguchi Y, editor. Advances in Cognitive Neurodynamics (III):. Springer Netherlands; 2013. p. 825–832.
